# Chronic myeloid and lymphocytic leukemias in the elderly: public health challenges in early detection and long-term care

**DOI:** 10.1097/MS9.0000000000004671

**Published:** 2026-01-09

**Authors:** Emmanuel Ifeanyi Obeagu, Aakib Rahman Parray

**Affiliations:** a Department of Biomedical and Laboratory Science, Africa University, Mutare, Zimbabwe; b Department of Molecular Medicine and Haematology, School of Pathology, Faculty of Health Sciences, University of the Witwatersrand, Johannesburg, South Africa; c Department of Psychology, Akal University, Talwandi Sabo, India

**Keywords:** chronic lymphocytic leukemia, chronic myeloid leukemia, early detection, elderly, long-term care

## Abstract

Chronic myeloid leukemia (CML) and chronic lymphocytic leukemia (CLL) predominantly affect older adults, presenting unique challenges in early detection and long-term management. The growing elderly population globally has intensified the public health burden of these chronic leukemias, particularly in low-resource settings. This narrative review synthesizes current evidence on the epidemiology, clinical features, early detection, and long-term care of CML and CLL in elderly populations, with a focus on public health implications and region-specific considerations. A structured literature search was conducted across PubMed, Scopus, Web of Science, and Google Scholar, including studies published from 2000 to 2025. Risk-based screening protocols, economic burden, and disparities in low-resource settings were emphasized. CML and CLL incidence increases with age, with median diagnosis ages of 67 and 70 years, respectively. Early detection is often delayed due to non-specific symptoms, limited molecular diagnostics, and sociocultural factors. Long-term management is complicated by comorbidities, treatment adherence challenges, and fragmented care systems. Evidence-based strategies, including risk-based screening, multidisciplinary care, and community-based interventions, improve outcomes. In low-resource settings such as Zimbabwe, disparities in diagnostic capacity, drug availability, and supportive care significantly affect disease outcomes. Economic analyses highlight that early detection and guideline-adherent monitoring are cost-effective, reducing complications and optimizing resource utilization. Effective management of CML and CLL in elderly populations requires integrated strategies combining early detection, personalized care, and health system strengthening. Tailored interventions addressing both clinical and socioeconomic determinants of health, particularly in low-resource environments, are essential to improve survival, quality of life, and cost efficiency.

## Introduction

Chronic myeloid leukemia (CML) and chronic lymphocytic leukemia (CLL) are two of the most prevalent types of leukemia diagnosed in adults^[1,2]^, with a notably higher incidence in the elderly population[3]. As global life expectancy increases, the number of older adults diagnosed with these chronic hematologic malignancies is expected to rise substantially[4]. This demographic shift poses significant challenges for health care systems, as older patients often present with distinct clinical features, comorbidities, and care requirements compared to younger individuals[5]. Understanding the public health implications of CML and CLL in the elderly is crucial for improving early diagnosis and ensuring effective long-term management[6]. Epidemiological data indicate that the median age at diagnosis for CML is approximately 65 years, while for CLL it is closer to 70 years^[7–9]^. Both diseases typically follow a chronic course but can vary widely in progression and response to treatment. Unlike acute leukemias, CML and CLL often present with subtle or nonspecific symptoms that may be overlooked or misattributed to aging or other chronic conditions^[10,11]^. This diagnostic ambiguity contributes to delays in identification, which can adversely impact treatment outcomes and survival rates in older patients.

The complexity of managing CML and CLL in elderly patients extends beyond the biological behavior of the disease[3]. Age-related physiological changes, such as decreased bone marrow reserve and altered drug metabolism, increase the risk of treatment-related toxicities and complications^[12,13]^. Furthermore, the presence of multiple comorbid conditions common in older adults complicates therapeutic decision-making and necessitates individualized treatment plans[14]. The delicate balance between controlling the malignancy and maintaining quality of life is a fundamental consideration in this population^[15,16]^. Early detection of CML and CLL in older adults remains a critical yet challenging goal for public health[6]. Many older patients do not exhibit overt symptoms at diagnosis, and routine screening for these leukemias is not a standard practice in most health care settings[17]. Additionally, limited awareness among primary care providers regarding the subtle clinical manifestations of chronic leukemias in the elderly may result in missed opportunities for timely referral and intervention[18]. Addressing these challenges requires improved education and enhanced screening protocols tailored to high-risk populations[19].

Long-term care for elderly patients with CML and CLL involves continuous disease monitoring, management of therapy-related side effects, and support for psychosocial needs[20]. Advances in targeted therapies, such as tyrosine kinase inhibitors (TKIs) for CML and novel immunotherapies for CLL, have significantly improved survival; however, prolonged treatment courses raise concerns about adherence, cumulative toxicity, and financial burden[21]. Comprehensive care models that integrate medical treatment with supportive services, including mental health and social support, are essential to optimize outcomes and maintain patient well-being^[22,23].^ Public health strategies must also consider disparities in health care access that disproportionately affect elderly populations, particularly in rural and low-resource settings. Socioeconomic factors, geographic barriers, and health care infrastructure limitations hinder timely diagnosis and consistent long-term care^[24,25]^. Policies aimed at reducing these disparities through improved resource allocation, community outreach, and telemedicine services can enhance equity in leukemia care among older adults[26]. The primary objective of this narrative review is to synthesize current evidence on CML and CLL in elderly populations, with a focus on public health challenges related to early detection, long-term management, and care coordination.

## Aim

This review aims to critically examine the public health challenges associated with CML and CLL in the elderly, with a particular focus on barriers to early detection and the complexities of long-term care management.

## Methods

This narrative review was conducted to synthesize the current literature on CML and CLL in elderly populations, with an emphasis on public health challenges in early detection and long-term care. While this is not a systematic review, we implemented a structured literature search to ensure transparency and comprehensiveness.

### Literature search strategy

A comprehensive search of the following electronic databases was performed: PubMed, Scopus, Web of Science, and Google Scholar. The search included articles published in English from January 2000 to October 2025. Search terms were developed to capture studies relevant to CML and CLL in elderly populations and included combinations of the following keywords:

“Chronic myeloid leukemia” OR “CML”

“Chronic lymphocytic leukemia” OR “CLL”

“elderly” OR “older adults” OR “geriatric”

“Epidemiology” OR “prevalence” OR “incidence”

“Early detection” OR “screening”

“Long-term care” OR “management” OR “public health”

Boolean operators (AND, OR) were used to refine search results. Reference lists of included studies and relevant review articles were manually screened to identify additional publications.

**HIGHLIGHTS**

- Aging immune systems delay leukemia detection, complicating diagnosis.
- Comorbidities hinder treatment choices and increase adverse outcomes.
- Limited geriatric oncology infrastructure affects care quality.
- Socioeconomic disparities impact early intervention.
- Lifelong monitoring strains public health resources.

### Inclusion and exclusion criteria

- Inclusion: peer-reviewed articles, clinical studies, observational studies, consensus guidelines, and review articles focused on CML or CLL in individuals aged ≥60 years, reporting epidemiology, clinical features, diagnostic strategies, management, or public health implications.

- Exclusion: studies primarily focused on pediatric populations, acute leukemias, non-English publications, or unrelated hematologic malignancies.

### Study selection and data extraction

Titles and abstracts were independently screened by two reviewers for relevance. Full-text articles meeting inclusion criteria were subsequently reviewed. Data were extracted on study characteristics, population demographics, disease epidemiology, diagnostic approaches, treatment modalities, and public health challenges. Discrepancies were resolved through discussion.

### Data synthesis

Given the narrative review design, findings were synthesized qualitatively. Emphasis was placed on summarizing epidemiological trends, early detection barriers, long-term care considerations, and evidence-based strategies for elderly populations, with attention to variations in resource availability across different settings.

### Ethical considerations

As this review relied on previously published literature, ethical approval was not required.

### Epidemiology and clinical features in the elderly

CML and CLL predominantly affect older adults[27], with incidence rates increasing significantly with age. Epidemiological data reveal that the median age of diagnosis for CML is approximately 65 years, whereas for CLL, it is around 70 years[9]. These malignancies account for a considerable proportion of hematologic cancers in the elderly population, reflecting demographic shifts and improved life expectancy worldwide^[4,28]^. The aging immune system, genetic alterations, and cumulative environmental exposures contribute to the heightened susceptibility to these leukemias in older individuals[29]. CML is characterized by the presence of the Philadelphia chromosome, resulting from a translocation between chromosomes 9 and 22, which leads to the BCR-ABL fusion gene and aberrant tyrosine kinase activity[30]. The disease generally progresses through three phases – chronic, accelerated, and blast crisis – with most elderly patients being diagnosed during the chronic phase[31]. Clinical manifestations may be mild or absent initially, often identified through routine blood tests showing leukocytosis or splenomegaly. Common symptoms in older adults include fatigue, night sweats, weight loss, and abdominal discomfort, although these are frequently nonspecific and easily attributed to aging or other comorbid conditions^[32,33]^.

CLL, the most common adult leukemia in Western countries, is characterized by the clonal expansion of mature B lymphocytes[34]. It typically follows an indolent course but can vary widely in clinical presentation and progression. Elderly patients with CLL often present with lymphadenopathy, fatigue, recurrent infections, or cytopenias[35]. Like CML, many cases are detected incidentally through abnormal blood counts. The clinical heterogeneity of CLL in older adults is influenced by genetic mutations, immune dysfunction, and comorbidities, which impact prognosis and treatment responsiveness[36]. Both CML and CLL in elderly patients are complicated by age-related physiological changes, including immune senescence, which affects immune surveillance and response to malignant cells^[37,38]^. Additionally, comorbid conditions such as cardiovascular disease, diabetes, and renal impairment are common and influence clinical decision-making. These factors contribute to a more complex disease course compared to younger populations, underscoring the need for age-specific diagnostic and management strategies[39].

CML and CLL predominantly affect older adults, with incidence and prevalence increasing with age. According to GLOBOCAN 2020, the global incidence of CML is approximately 1–2 cases per 100 000 population per year, while CLL incidence ranges from four to six per 100 000, with higher rates in Western countries and lower reported rates in sub-Saharan Africa, likely reflecting underdiagnosis and limited registry coverage. Data from the SEER database in the United States indicate a median age at diagnosis of 67 years for CML and 70 years for CLL, with age-adjusted prevalence steadily rising due to improved survival with targeted therapies. National cancer registries in high-income countries further reveal a shift toward increased chronic management, underscoring the growing burden on health care systems^[34,35]^.

The economic impact of these leukemias is substantial. Studies have estimated the annual direct medical costs for CML patients on TKIs to range between $40 000 and $70 000 per patient, while costs for CLL management, particularly with novel monoclonal antibodies and combination regimens, may exceed $60 000 annually in high-income settings. Early detection and risk-based monitoring have been shown to be cost-effective, reducing hospitalizations for advanced disease, preventing complications, and optimizing therapy utilization. For example, modeling studies demonstrate that implementing guideline-recommended CBC monitoring and molecular testing for high-risk older adults can decrease overall health care expenditures while improving clinical outcomes^[36,37]^. These epidemiological and economic data provide a foundation for evidence-based discussions of screening strategies, resource allocation, and health care planning, particularly in settings where diagnostic and therapeutic resources are limited. They highlight the importance of prioritizing early detection and cost-efficient monitoring to mitigate the clinical and financial burden of chronic leukemias in elderly populations^[38,39]^.

### Barriers to early detection

Early detection of CML and CLL in the elderly is critical for improving treatment outcomes and survival[6]. However, multiple barriers impede timely diagnosis in this population. One major challenge is the often subtle and nonspecific clinical presentation of these leukemias in older adults. Symptoms such as fatigue, mild anemia, night sweats, or weight loss are frequently attributed to normal aging or other chronic illnesses, leading to delayed clinical suspicion and diagnostic evaluation^[40,41]^. Another significant barrier is the lack of standardized screening protocols for CML and CLL, especially in asymptomatic elderly individuals^[42,43]^. Unlike some solid tumors where routine screening is established, chronic leukemias are typically diagnosed after incidental findings during blood tests or when symptoms worsen[44]. This absence of systematic screening contributes to diagnostic delays and can result in presentation at more advanced disease stages, which are harder to manage effectively[45].

Health care provider factors also play a role in delayed detection[20]. Primary care physicians and non-hematology specialists may have limited awareness of the subtle signs and early laboratory abnormalities associated with chronic leukemias in older patients[46]. This knowledge gap can result in under-referral to hematology specialists and missed opportunities for early intervention. Additionally, time constraints during clinical encounters and competing health care priorities for multiple comorbidities often reduce focus on possible hematologic malignancies[47]. Socioeconomic and systemic barriers further complicate early detection efforts. Elderly patients, particularly those in rural or underserved areas, may face limited access to health care services, diagnostic facilities, and specialist consultations[48]. Transportation challenges, financial constraints, and fragmented health care systems can delay or prevent appropriate evaluation[49]. Moreover, disparities related to education level, health literacy, and cultural beliefs influence health-seeking behaviors and willingness to undergo diagnostic procedures[50]. Psychosocial factors such as fear of diagnosis, fatalistic attitudes toward cancer, and stigma can also inhibit timely presentation to health care providers^[51,52]^. Elderly individuals may prioritize other health issues or accept symptoms as part of aging rather than seek medical advice[53]. This delay in help-seeking behavior reduces the likelihood of early detection and intervention (Table 1).

**Table 1:** Barriers to early detection of CML and CLL in elderly populations

Barrier category	Description	Impact on early detection	Potential mitigation strategies
Clinical presentation	Non-specific symptoms (fatigue, weight loss, mild anemia) often attributed to normal aging.	Delayed recognition and referral to hematology specialists.	Educate primary care providers and patients on early warning signs; implement routine CBC review for older adults.
Diagnostic access	Limited availability of molecular testing (BCR-ABL1 for CML, flow cytometry for CLL), especially in rural or low-resource areas.	Delays in confirmatory diagnosis and therapy initiation.	Expand laboratory infrastructure; use regional referral centers; subsidize molecular testing.
Health care system limitations	Fragmented care pathways, insufficient trained personnel, and long wait times.	Late-stage presentation and reduced treatment effectiveness.	Develop coordinated care networks; train primary care and community health workers in early leukemia recognition.
Sociocultural factors	Lack of awareness among older adults; stigma associated with cancer; misattribution of symptoms to aging.	Reduced health-seeking behavior and delayed diagnosis.	Community awareness campaigns; patient education programs; culturally sensitive outreach.
Economic barriers	High costs of diagnostic tests and travel to specialized centers.	Financial constraints prevent timely evaluation.	Implement subsidized testing programs; integrate screening into routine primary care visits.
Policy and guideline gaps	Absence of standardized risk-based screening protocols for elderly populations in many countries.	Variability in detection rates and late presentation.	Adopt and implement risk-based screening protocols recommended by NCCN and ESMO; integrate geriatric assessments in routine practice.

CBC, complete blood count; CLL, chronic lymphocytic leukemia; CML, chronic myeloid leukemia; ESMO, European Society for Medical Oncology; NCCN, National Comprehensive Cancer Network.

### Long-term care challenges

Long-term management of CML and CLL in elderly patients presents a complex array of challenges that extend beyond disease control^[54,55]^. Aging-related physiological changes, including reduced organ function, altered drug metabolism, and immunosenescence, significantly influence both treatment efficacy and toxicity. These factors necessitate careful therapeutic planning and dose adjustments to balance disease suppression with preservation of quality of life[56]. Polypharmacy is a common concern in elderly patients, who often manage multiple comorbidities such as cardiovascular disease, diabetes, and renal impairment alongside leukemia^[57,58]^. The risk of drug–drug interactions increases with the complexity of treatment regimens, including TKIs for CML and targeted immunotherapies or chemoimmunotherapy for CLL[59]. Such interactions can exacerbate side effects, reduce treatment adherence, and complicate clinical monitoring[60].

Adherence to long-term therapy is another critical challenge^[61,62]^. Chronic leukemias generally require sustained treatment over years or even lifelong, which can be burdensome for older adults due to side effects, cognitive decline, or social support limitations[63]. Nonadherence may lead to disease progression or resistance, emphasizing the need for continuous patient education, psychosocial support, and adherence monitoring[64]. Furthermore, elderly patients face a heightened risk of treatment-related complications such as infections, cytopenias, and secondary malignancies due to both the disease and its therapies[65]. Immunosenescence compounds susceptibility to infections and reduces vaccine responsiveness, requiring vigilant preventive measures and prompt management of complications[66]. The management of adverse effects often demands a multidisciplinary approach involving hematologists, geriatricians, pharmacists, and nursing staff^[67–69]^.

Psychosocial issues, including social isolation, depression, and caregiver burden, profoundly impact long-term care outcomes[70]. Many elderly patients rely on informal caregivers who may lack adequate resources or training, increasing the risk of suboptimal care and hospital readmissions. Comprehensive support systems and integration of community services are vital to address these needs and promote holistic care. Financial toxicity is an additional barrier, as long-term treatment and supportive care for CML and CLL can be costly[71]. Elderly patients on fixed incomes may struggle with medication costs, frequent clinic visits, and ancillary expenses, which can adversely affect adherence and quality of life[72]. Public health policies aimed at reducing out-of-pocket expenses and improving insurance coverage are essential components of sustainable long-term care (Table 2)[73].

**Table 2:** Long-term care challenges in elderly patients with CML and CLL

Challenge category	Description	Impact on patient outcomes	Potential mitigation strategies
Comorbidities	High prevalence of cardiovascular disease, diabetes, renal impairment, and other chronic conditions.	Complicates treatment selection, increases risk of adverse events, and may limit therapy options.	Integrate geriatric and multidisciplinary assessments; personalize treatment plans based on comorbidity profiles.
Treatment adherence	Polypharmacy, cognitive decline, and side effects of therapy (e.g., TKIs, monoclonal antibodies).	Reduced adherence leads to suboptimal therapeutic response and risk of disease progression.	Provide patient education, simplified dosing schedules, caregiver support, and telehealth follow-up.
Health care access and continuity	Limited availability of specialists, fragmented care systems, and long travel distances in rural areas.	Interruptions in monitoring, delayed management of complications, and increased hospitalization rates.	Establish community-based clinics, nurse-led monitoring programs, and coordinated referral networks.
Socioeconomic factors	Financial constraints, lack of insurance, and limited social support.	Increased treatment interruptions and reduced access to supportive care services.	Implement subsidized therapy programs, social worker support, and community resource linkage.
Psychosocial challenges	Anxiety, depression, caregiver burden, and social isolation common among elderly patients.	Decreased quality of life, lower adherence, and impaired decision-making.	Incorporate mental health services, counseling, support groups, and caregiver education.
Monitoring and surveillance	Need for frequent blood counts, molecular testing, and assessment of treatment toxicity.	Logistical challenges may reduce monitoring compliance, delaying detection of complications or disease progression.	Develop structured follow-up protocols, utilize telemedicine, and engage community health workers in routine monitoring.

CLL, chronic lymphocytic leukemia; CML, chronic myeloid leukemia; TKIs, tyrosine kinase inhibitors.

### Public health strategies

Addressing the unique challenges of CML and CLL in the elderly requires coordinated public health strategies aimed at improving early detection, enhancing long-term care, and reducing health disparities[74]. Central to these efforts is the development of targeted awareness campaigns that educate both health care providers and older adults about the subtle symptoms and risk factors of these leukemias. Enhancing knowledge can prompt earlier diagnostic evaluation and referral to specialists, ultimately improving prognosis[75]. Screening and diagnostic accessibility represent crucial components of effective public health intervention[76]. While routine population-wide screening for CML and CLL is not currently standard practice, risk-based screening strategies could be integrated into primary care for older adults, particularly those with predisposing factors or unexplained hematologic abnormalities[77]. Increasing access to affordable and timely diagnostic services in community and rural settings through mobile clinics, telehealth, and decentralized laboratory networks can mitigate geographic and socioeconomic barriers^[78,79]^.

The implementation of multidisciplinary care models is essential for managing the complex needs of elderly leukemia patients. Such models promote collaboration among hematologists, geriatricians, primary care providers, pharmacists, social workers, and mental health professionals[80]. Public health policies should support the integration of these teams within health care systems, ensuring comprehensive assessment and individualized care plans that consider comorbidities, functional status, and psychosocial factors[3]. Health systems strengthening through provider training and capacity building is another priority[81]. Continuing education programs focusing on geriatric oncology and hematology can equip health care workers with skills to recognize early signs, manage therapy complications, and communicate effectively with elderly patients and their families[82]. Enhanced provider competency can reduce delays in diagnosis and improve adherence to evidence-based treatment protocols.

Addressing social determinants of health, including transportation, socioeconomic status, and health literacy, is vital to reduce disparities in leukemia care[83]. Public health initiatives that link patients with community resources, provide caregiver support, and facilitate health navigation can improve treatment adherence and quality of life. Expanding insurance coverage and financial assistance programs to offset treatment costs will also alleviate economic burdens on elderly patients[9]. Fostering research and data collection focused on elderly populations with CML and CLL is necessary to inform evidence-based policies and interventions[63]. Surveillance systems that capture age-specific incidence, treatment outcomes, and barriers to care can guide resource allocation and program development[83]. Engaging patients and caregivers in research initiatives will ensure that strategies are patient-centered and culturally appropriate (Table 3)^[81–84]^.

**Table 3:** Public health strategies for improving detection and long-term care in elderly leukemia patients

Strategy category	Description	Feasibility in low-resource settings	Potential impact on outcomes
Risk-based screening	Targeted CBC monitoring, flow cytometry, and molecular testing (BCR-ABL1 for CML, clonal B-cell assessment for CLL) in high-risk older adults.	Moderate; requires basic lab infrastructure and training.	Enables earlier diagnosis, reduces progression to advanced disease, and improves survival.
Multidisciplinary Care models	Integration of hematology, geriatrics, nursing, pharmacy, and social work in patient management.	High; scalable with existing primary care teams and telehealth support.	Optimizes therapy selection, reduces adverse events, and improves quality of life.
Community-based Interventions	Nurse-led follow-up clinics, mobile health units, patient education, and caregiver support programs.	High; can be implemented with limited resources.	Improves adherence, reduces hospitalizations, and enhances psychosocial support.
Access to targeted therapies	Provision of TKIs for CML and monoclonal antibodies for CLL through subsidized programs, donations, or insurance coverage.	Moderate; dependent on government or NGO support.	Increases treatment availability, reduces disease progression, and enhances survival.
Health system Strengthening	Expansion of laboratory capacity, development of referral networks, and implementation of national leukemia registries.	Variable; requires policy support and investment.	Improves diagnostic accuracy, data collection, and resource allocation.
Patient and caregiver education	Awareness campaigns on early symptoms, adherence strategies, and supportive care resources.	High; low-cost, adaptable to local contexts.	Enhances early presentation, adherence, and engagement with care.
Economic and policy interventions	Subsidized testing, therapy access programs, and incorporation of leukemia care into national health plans.	Moderate; dependent on political will and funding.	Reduces financial barriers, improves equity, and ensures sustainability of care.

CBC, complete blood count; CLL, chronic lymphocytic leukemia; CML, chronic myeloid leukemia; NGO, non-governmental organization; TKI, tyrosine kinase inhibitor.

### Region-specific considerations: Zimbabwe and low-resource settings

CML and CLL present unique challenges in low-resource settings such as Zimbabwe, where health care infrastructure, access to diagnostics, and availability of targeted therapies are often limited. Epidemiological data from Zimbabwe and neighboring countries are sparse, reflecting both underdiagnosis and limited cancer registries, which complicates efforts to quantify disease burden accurately. Consequently, many patients are diagnosed at advanced stages, often following the emergence of severe symptoms, including profound cytopenias or organomegaly, which could have been mitigated by earlier detection[80]. Access to molecular diagnostics – critical for confirming CML via BCR-ABL1 testing or for identifying prognostic markers in CLL – is often restricted to urban tertiary hospitals, leaving rural populations underserved. This geographic disparity delays initiation of therapy and may lead to suboptimal outcomes. Even when diagnosis is confirmed, access to targeted therapies such as TKIs for CML or novel monoclonal antibodies for CLL is frequently limited by high cost, inconsistent drug supply, or lack of health insurance coverage. Patients may rely on public hospital programs with intermittent drug availability, which can compromise adherence and increase the risk of disease progression or resistance[81].

Supportive care, including management of treatment-related toxicities, transfusion support, and infection prophylaxis, is similarly constrained. Resource limitations often necessitate prioritization of care, leaving elderly patients – who may have comorbidities such as hypertension, diabetes, or renal impairment – particularly vulnerable. Additionally, psychosocial support systems, including patient education and community-based care networks, are underdeveloped, further complicating long-term management and adherence[82]. To address these disparities, region-specific strategies are essential. Strengthening primary care and laboratory capacity, expanding access to molecular diagnostics, and implementing subsidized or donation-based programs for targeted therapies could improve outcomes. Community health worker programs and nurse-led follow-up clinics can provide ongoing monitoring and patient education, bridging gaps in rural and underserved areas. Furthermore, establishing national leukemia registries and fostering collaborations with international cancer networks would enhance data collection, resource allocation, and research tailored to local needs[83].

### Risk-based screening and early detection protocols in older adults

Early detection of CML and CLL in elderly populations remains a critical determinant of long-term outcomes, yet routine population-based screening is not currently recommended. Instead, risk-based screening protocols guided by clinical and laboratory indicators are emphasized in international guidelines, including the National Comprehensive Cancer Network (NCCN) and the European Society for Medical Oncology (ESMO)^[78,79].^ For CML, early identification primarily relies on the recognition of hematologic abnormalities such as leukocytosis, thrombocytosis, or unexplained splenomegaly. NCCN guidelines recommend that adults, particularly those over 60, who present with persistent unexplained leukocytosis, undergo complete blood counts (CBC) with differential, followed by molecular testing for BCR-ABL1 if abnormal findings are detected. ESMO similarly advocates for risk-based molecular testing in patients with clinical suspicion, emphasizing that early detection allows prompt initiation of TKIs, which dramatically improve survival and reduce progression to advanced disease phases[80].

For CLL, screening is guided by incidental laboratory findings, particularly lymphocytosis on routine CBCs. Both NCCN and ESMO recommend flow cytometry to confirm clonal B-cell populations when absolute lymphocyte counts are elevated in older adults, especially those with additional risk factors such as family history of hematologic malignancies or recurrent infections. Follow-up intervals are stratified based on disease stage and risk profile; asymptomatic patients with low-risk CLL may be monitored every 3–12 months, whereas higher-risk patients require more frequent evaluation^[81,82]^. Risk-based strategies also incorporate frailty assessments and comorbidity evaluations to individualize the frequency and intensity of monitoring in elderly patients. Integrating geriatric assessments with laboratory-based screening ensures that early detection efforts balance clinical benefit with patient safety and resource utilization[83].

In low-resource or rural settings, simplified protocols emphasizing CBC monitoring and targeted referrals for molecular confirmation have been validated to improve early detection rates without imposing excessive health care burdens. These strategies demonstrate that structured, risk-based screening – guided by internationally recognized standards – can optimize timely diagnosis, enable personalized treatment initiation, and ultimately improve outcomes for older adults with chronic leukemias[84].

### Recommendations

1. Enhance Awareness and Education: Implement targeted educational programs for primary care providers and older adults to improve recognition of early symptoms and risk factors associated with CML and CLL. Utilizing community outreach and digital platforms can broaden reach, especially among underserved populations.
2. Develop Risk-Based Screening Protocols: Promote research and pilot programs to establish evidence-based, risk-adapted screening strategies for elderly populations at higher risk of CML and CLL. Routine blood count monitoring during geriatric assessments can serve as a practical approach to flagging potential cases for further evaluation.
3. Strengthen Multidisciplinary Care Models: Encourage health care systems to adopt integrated care teams comprising hematologists, geriatricians, pharmacists, social workers, and mental health specialists. Such collaboration ensures holistic management addressing medical complexities and psychosocial needs of elderly leukemia patients.
4. Improve Access to Diagnostic and Treatment Services: Expand diagnostic facilities and specialist services in rural and low-resource areas via telemedicine, mobile clinics, and decentralized laboratories. Financial support programs and insurance coverage enhancements are vital to reduce out-of-pocket expenses and ensure equitable care access.
5. Promote Patient and Caregiver Support Programs: Establish support networks offering education, counseling, and respite care for elderly patients and their caregivers. Empowering caregivers through training and resources improves adherence, reduces hospitalizations, and enhances quality of life.
6. Integrate Social Determinants of Health into Care Planning: Systematically assess factors such as socioeconomic status, transportation, and health literacy during clinical encounters. Link patients to community resources and social services to address these barriers comprehensively.
7. Advance Research Focused on the Elderly: Prioritize funding for studies investigating age-specific disease biology, treatment responses, and long-term outcomes in elderly patients with CML and CLL. Encourage inclusion of diverse elderly populations to ensure generalizability of findings.
8. Policy Advocacy: Engage policymakers to recognize chronic leukemias in the elderly as a public health priority. Advocate for allocation of resources toward education, screening, multidisciplinary care, and support services tailored to this demographic.

## Conclusion

CML and CLL in elderly populations represent a growing public health challenge, particularly in low-resource settings. Early detection is often hindered by limited access to molecular diagnostics, non-specific clinical presentations, and gaps in risk-based screening, while long-term care is complicated by comorbidities, polypharmacy, and fragmented health care systems.

Despite these challenges, advances in targeted therapies, evidence-based monitoring protocols, and multidisciplinary care models offer significant opportunities to improve outcomes. Implementing structured risk-based screening, integrating geriatric assessment into treatment planning, and developing community-based support systems can enhance early diagnosis, optimize therapy adherence, and improve quality of life for older adults. Region-specific considerations, such as disparities in Zimbabwe and similar low-resource environments, underscore the need for tailored interventions, including expanded diagnostic access, subsidized treatment programs, and strengthened health care infrastructure. Policy initiatives that support sustainable access to care, along with robust national registries, are essential to monitor disease trends and guide resource allocation.
